# Recent Advances in Electrochemical Biosensors Based on Enzyme Inhibition for Clinical and Pharmaceutical Applications

**DOI:** 10.3390/s18010164

**Published:** 2018-01-09

**Authors:** Loubna El Harrad, Ilhame Bourais, Hasna Mohammadi, Aziz Amine

**Affiliations:** Laboratory of Process Engineering & Environment, Faculty of Sciences and Technology, Hassan II University of Casablanca, Mohammadia B.P.146, Morocco; elharrad.l@hotmail.fr (L.E.H.); ilhame_b@yahoo.com (I.B.); hasna2001fr@yahoo.fr (H.M.)

**Keywords:** electrochemical biosensors, enzyme inhibition, diagnosis of inhibition, drug, disease

## Abstract

A large number of enzyme inhibitors are used as drugs to treat several diseases such as gout, diabetes, AIDS, depression, Parkinson’s and Alzheimer’s diseases. Electrochemical biosensors based on enzyme inhibition are useful devices for an easy, fast and environment friendly monitoring of inhibitors like drugs. In the last decades, electrochemical biosensors have shown great potentials in the detection of different drugs like neostigmine, ketoconazole, donepezil, allopurinol and many others. They attracted increasing attention due to the advantage of being high sensitive and accurate analytical tools, able to reach low detection limits and the possibility to be performed on real samples. This review will spotlight the research conducted in the past 10 years (2007–2017) on inhibition based enzymatic electrochemical biosensors for the analysis of different drugs. New assays based on novel bio-devices will be debated. Moreover, the exploration of the recent graphical approach in diagnosis of reversible and irreversible inhibition mechanism will be discussed. The accurate and the fast diagnosis of inhibition type will help researchers in further drug design improvements and the identification of new molecules that will serve as new enzyme targets.

## 1. Introduction

The living organisms are the site of many reactions driven by enzymes. The aberrant activity of one of these enzymes can result in disruption of the normal biological process leading to the occurrence of diseases. One of the main therapeutic approaches in the treatment and prevention of several diseases such as cancer, AIDS, Alzheimer’s and Parkinson’s diseases, depression and hyperthyroidism is based on the use of drugs that act on enzyme activity, especially as inhibitors [1,2,3,4,5]. The implication of many enzymes such as acetylcholinesterase, xanthine oxidase and cytochrome oxidase in drug therapy is reported in the literature. The mechanism of enzyme inhibition by drugs can be either reversible or irreversible. Reversible inhibition is the most common mechanism observed (Table 1). Therefore, the study of enzyme inhibition induced by drugs, the screening of inhibitors and the monitoring of their toxicity are necessary.

Drugs are considered as xenobiotics, so they are metabolized by enzymatic reactions into one or more other active/inactive pharmacological compounds [6,7]. The formation of toxic metabolites is also possible [8]. Many tissues can perform this transformation (e.g., liver, skin, lung, kidney and intestine) [9]. Nevertheless, the main biotransformation site is represented at the hepatic level by the microsomal enzymes [10]. For instance, cytochrome P450 (CYP) are predominant enzymes present in the liver and play a crucial role against xenobiotics [11,12]. CYP2 A13 isoform acts on nicotine by producing tobacco-specific carcinogens [13]. Thus, the inhibition of the activity of this enzyme using some drugs aims to reduce the initiation of lung cancer in smokers or people exposed to nicotine. In another case, the inhibition of HIV protease induces the reduction of the process of multiplication of the HIV virus in the body. Therefore, HIV protease inhibitors are prescribed as antiretroviral drugs [14].

In analytical biochemistry, researchers use in vitro tests to determine the influence of drugs on enzymes to test their effectiveness in single use and sometimes in drug mixtures. Enzymatic inhibition studies are used to follow pharmacokinetic changes that may affect drug effectiveness and safety, especially if they have a low therapeutic index. Furthermore, in silico method showed a great interest for the development of new drugs by predicting the metabolic pathways of physiologically active substances and their interactions on a molecular level [15].

**Figure 1:**
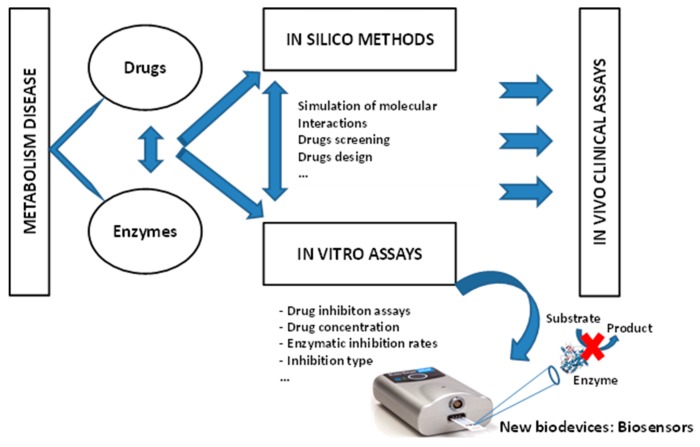
Relationship between in silico prediction and new analytical tools and their importance in drug therapy as preliminary assays before proceeding to in vivo tests.

Development of new analytical tools for simultaneously monitoring of drug concentration and enzymatic inhibition rates for in vitro experiments is necessary before proceeding to in vivo assays, especially for testing newly designed drugs (Figure 1). Accordingly, rapid and effective methods for the determination of drugs are required in many areas, including clinical chemistry, pharmaceutical, as well as nutrition.

Over the last decade, considerable interest has been focused on enzyme-based electrochemical biosensing systems as simple and effective tools for analytical investigations as an alternative to conventional methods. The utility of these bio-devices comes from their simple sensing systems to analyze the substrate, the generated product, or even the screening and measurement of other compounds such as drugs that act as inhibitors of enzymatic activity. Remarkable developments in enzymatic electrochemical biosensors designed for screening of therapeutic compounds with required analytical performances have been reported [16,17,18,19]. These electrochemical biosensors use many enzymes and the most studied path is that of drug targets in the metabolic pathway of living organisms. These drug targets act as enzyme inhibitors; consequently, the inhibitor concentration affecting the rate of this enzymatic transformation can be measured. Electrochemical biosensors perform measurements using small sample volumes, low concentrations of biological components and sometimes miniaturized analytical devices [20,21,22,23,24].

The importance of using enzymes for analytical purposes is due to their numerous properties as precision in the measurement of enzymatic reaction rates, the sensitivity towards the substrate due to the ‘key-lock’ configuration, the suitability for the needs and the wide diversity of analytical enzymes available on a large-scale. In clinical and pharmaceutical fields, enzymatic electrochemical biosensors based on inhibition are very promising and offer numerous advantages such as robustness, portability and cost-effectiveness.

Over the past years, our group has published reviews dealing with electrochemical biosensors based on enzyme inhibition for the determination of different compounds such as pesticides, food contaminants and drugs [25,26]. However, in this review, we focus our work on the research activities dedicated to pharmaceutical and clinical analysis carried out over the last decade with electrochemical biosensing systems based on enzyme inhibition. The numerous enzymes used for electrochemical biosensors design, transducers modification and inhibitors behaviors are fully discussed in this work.

## 2. Diagnosis of Inhibition Type

Enzymatic inhibition is considered as the key point in clinical applications and drug therapy. Indeed, several drugs such as neostigmine, donepezil, allopurinol, methimazole and moclobemide induce the inhibition of target enzymes involved in various diseases [42,43,44]. Accordingly, sensitive biosensors based on enzyme inhibition are highly required to monitor these drugs.

**Scheme 1:**
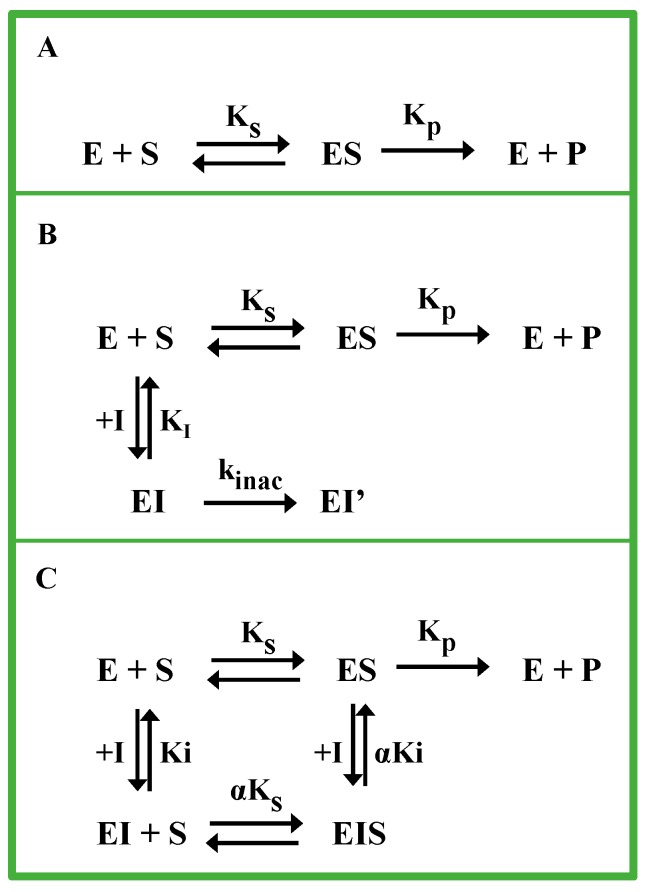
Kinetics of non-inhibitive enzymatic reaction (**A**), irreversible enzyme inhibition (**B**) and reversible enzyme inhibition (**C**).

**Figure 2:**
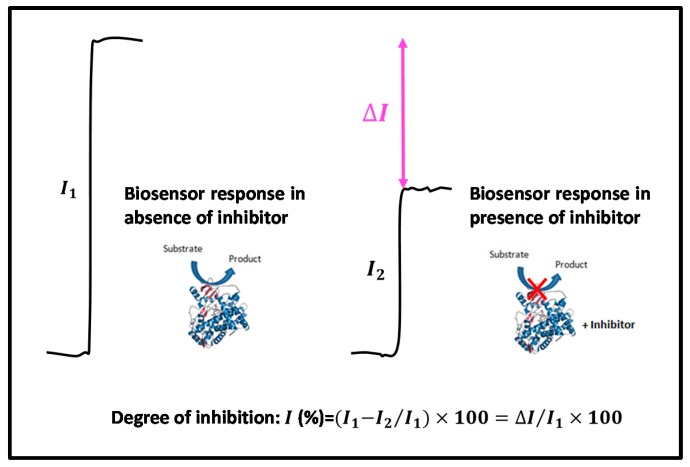
Degree of inhibition determination using enzyme-based electrochemical biosensor.

The response of biosensor based on enzyme inhibition is recorded prior addition of inhibitor ($I_{1}$) and after addition of inhibitor ($I_{2}$) thereafter, the degree of inhibition (I%) can be calculated (Figure 2). The enzymatic reaction in absence of inhibitor is illustrated in Scheme 1A. The enzymatic inhibition mechanism can be reversible or irreversible. In irreversible inhibition, the inhibitor binds covalently to the active site of the enzyme resulting the permanent inactivation of the enzyme [25,26,45,46]. Consequently, the enzyme losses its original activity that cannot be recovered (Scheme 1B).

On the other hand, the reversible inhibition is characterized by an equilibrium between enzyme and inhibitor and is divided into four different types: competitive, uncompetitive, non-competitive and mixed inhibition. The common types of inhibition can be regarded as particular cases of mixed type, as reported in Scheme 1C. Indeed, mixed type can include competitive, noncompetitive and uncompetitive types by assuming α >>1, α = 1 and α <<1, respectively. In competitive inhibition, both the substrate and the inhibitor compete for the same active site of the enzyme. Meanwhile, in uncompetitive inhibition, the substrate binds to the complex enzyme-inhibitor. In the non-competitive inhibition, both substrate and inhibitor bind to different sites of the enzyme with the same affinity or with different affinity as in the case of mixed inhibition [47].

Recently Amine et al., [48] proposed a novel graphical approach based on the degree of inhibition for the diagnosis of reversible inhibition type and the determination of $I_{50}$ which represents the concentration of the inhibitor leading to 50% of inhibition.

It is worth remembering that in the case of immobilized enzyme, it is easy to distinguish between reversible and irreversible inhibition by simple washing steps [49,50]. However, when dealing with the enzyme in solution, it became complicated to differentiate between them.

**Figure 3:**
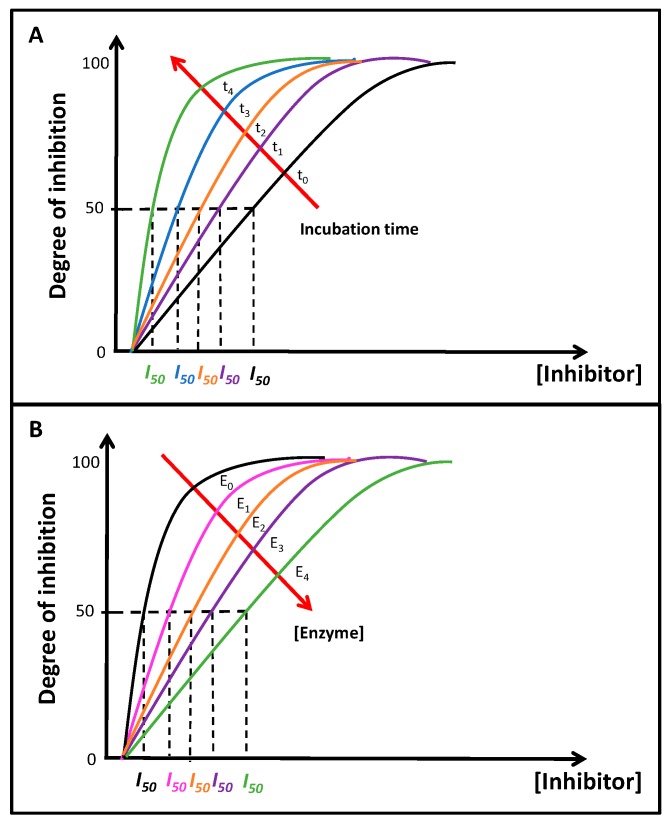
Effect of incubation time (**A**) and enzyme concentration (**B**) on $I_{50}$ in the case of irreversible inhibition.

Taking into account that irreversible inhibition requires time of incubation and low concentrations of the enzyme [51], we propose in this work another graphical approach based on the degree of inhibition and determination of $I_{50}$. This approach allows the estimation of $I_{50}$ directly from the plot, which can be employed for distinction between different types of inhibition. When varying the incubation time of the inhibitor with the enzyme, the typical plot of the degree of inhibition versus the inhibitor concentration at fixed concentration of substrate is depicted in Figure 3A.

**Figure 4:**
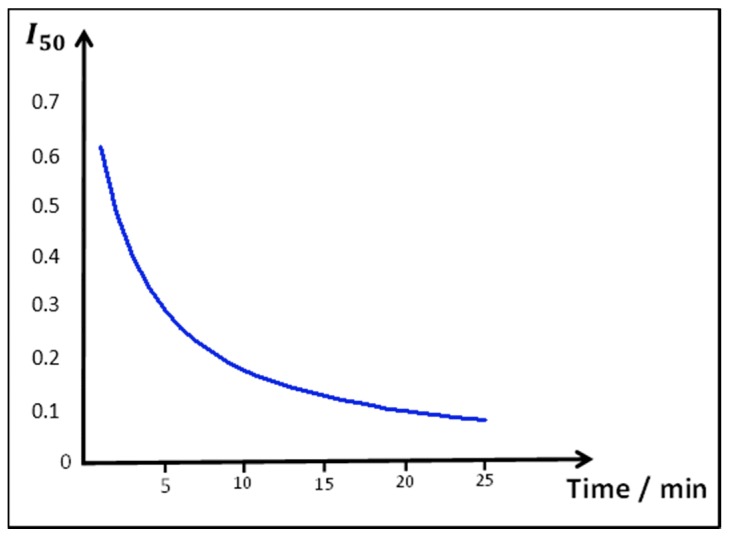
Variation of $I_{50}$ in function of time in case of irreversible inhibition. The curve was plotted using the equation of I50=0.69×Ki((Kinac×t)+0.69) and the following parameters were used: $K_{i}=0.84\;µM$, $K_{inac}=0.25\;\min^{-1}$.

From the hyperbolae curves in Figure 3A, the change in the obtained $I_{50}$ at different time of incubation can be used to distinguish between irreversible and reversible inhibition. Indeed, in irreversible inhibition, $I_{50}$ decreases when incubation time increases and the curves shift to the left (Figure 3A), meanwhile, $I_{50}$ remains the same in reversible inhibition. The inhibition type can be further determined by varying different enzyme concentrations and plotting the degree of inhibition against the concentration of inhibitor as shown in Figure 3B. In irreversible inhibition, $I_{50}$ increases proportionally with increasing enzyme concentration and the curves shift to the right (Figure 3B). However, reversible inhibition is not affected by enzyme concentration and consequently $I_{50}$ does not change. The incubation time is a very important parameter in irreversible inhibition since the degree of inhibition increases and $I_{50}$ decreases with increasing time of incubation. However, an over incubation time it’s not needed to obtain smaller $I_{50}$ because after a period of incubation, $I_{50}$ stay unvaried even with longer incubation time as calculated from the original equation of Kitz and Wilson of irreversible inhibition [52] and as showed in typical plot in Figure 4. Therefore, for irreversible inhibition $I_{50}$ is small when an appropriate incubation time and small amount of enzyme are used. However, the disadvantage of using too small amount of enzyme is that the detection of inhibition can be hardly observed and be unable to reach a small detection limit which will require a very high sensitive methods for detection.

**Figure 5:**
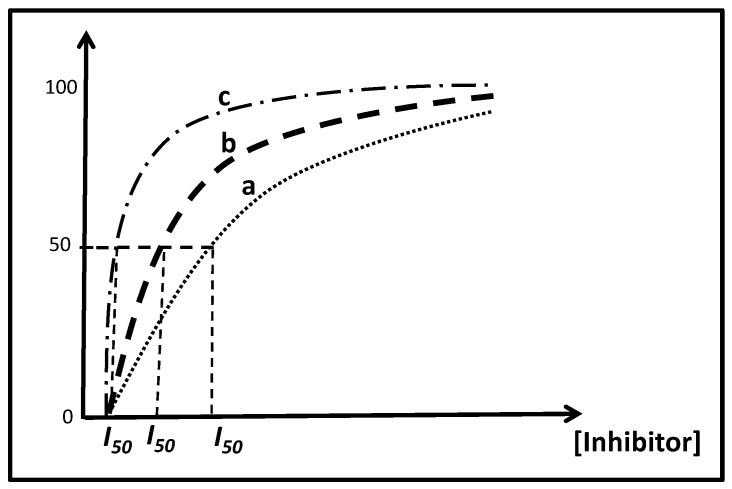
Effect of concentration of substrate on $I_{50}$ in case of reversible inhibition (curve **a**) competitive, (curve **b**) non-competitive and (curve **c**) uncompetitive.

In the other hand, this approach based on the degree of inhibition can be employed to reversible inhibition as described previously by Amine et al. [48] to distinguish between competitive, uncompetitive and non-competitive inhibition. For the diagnosis of inhibition type, the degree of inhibition was plotted against the inhibitor concentration using a fixed concentration of substrate [S], and a calibration curve was obtained (Figure 5 curve b). Indeed, in competitive inhibition, when the concentration of substrate [S] increases, $I_{50}$ increases too and the curve shift to the right (Figure 5 curve a), meanwhile in uncompetitive inhibition, when the concentration of substrate [S] increases $I_{50}$ decreases and the curve shift to the left (Figure 5 curve c). However, in non-competitive inhibition, $I_{50}$ is not affected by the concentration of substrate and the curve remains the same (Figure 5 curve b). Accordingly, $I_{50}$ is affected by the concentration of substrate and it’s not unvaried as the inhibition constant $K_{i}$.

Thus, this graphical approach based on plotting degree of inhibition versus inhibitor concentration at various times of incubation, enzyme and substrate concentrations is an easy and fast method that can be used to distinguish between irreversible and reversible inhibition and to determine different types of reversible inhibition with the advantage of the estimation of $I_{50}$ graphically.

## 3. Biosensor Design

**Figure 6:**
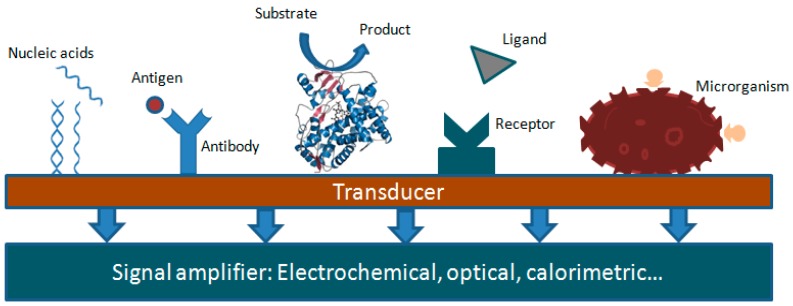
Schematic presentation of different biosensors based on numerous bio-recognition elements.

Biosensors can be classified according to several parameters [53]. Based on the type of molecular recognition (bioreceptor), they can be divided into enzymatic biosensors (with an enzyme as a bioreceptor), immuno or microbial biosensors, etc. (Figure 6), Secondly, by the type of associated transducer, we distinguish between electrochemical biosensors, optical biosensors, calorimetric biosensors, etc., Finally, according to the species detected, as substrate or inhibitors. They provide wide applications in detection of chemical and biological targets [54,55]. 

Biosensors are analytical tools offering quantitative or semi-quantitative analytical information by means of a biological recognition element in contact with a suitable transducer. In the fabrication of biosensors, the choice of an appropriate matrix and the immobilization procedure are the most important factors affecting the biosensor analytical performances [56].

Enzymes can be immobilized onto the surface of a transducer via several immobilization techniques such as physical adsorption, covalent binding, entrapment and covalent cross-linking [56,57,58]. The arrangement of biosensor affects the direct electron transfer between the electrode and electro active centers of biomolecules. From 1990 until now, several studies that focus on biomolecule immobilization using co-adsorbent stabilizing agents have been carried out to improve the electrochemical transfer between the electrode surface and the electroactive centers of the biological species and to overcome the problem of denaturation and biomolecules poor stability. Physical adsorption, entrapment and covalent binding methods remain the most preferred methods in the immobilization of an enzyme.

On the other hand, increasing attention is being given to the development of new biosensors applying nanostructured materials, such as carbon nanotubes and metal nanoparticles as modifiers of the electrode surface [45,59]. Nanomaterials have specific properties and features useful for the modification of various transducers such as screen-printed electrodes, which can change the surface of the electrode and make it more conducting [60]. In electrochemical investigations, it is very difficult to achieve the direct electron transfer of an enzyme without any modification of the electrode surface. One of the most promising technologies in transducers elaboration involves the use of novel materials with a suitable nanostructured surface [61,62].

The design of these biological sensing systems regarding different developed materials has been reported. Metallic or non-metallic transducers have been used to ensure similar physiological conditions for enzyme metabolism. Many materials have been used to modify the electrode surface to enhance the sensitivity of enzyme-based biosensors by increasing the effective surface area of the electrode to load large amounts of enzyme [63].

Carbon nanotubes (CNTs) and carbon nanofibers (CNFs) are some of the novel nanomaterial components used in transducer design to improve the electrical signals from enzymes to the electrode [64,65].

Recently, electrochemical biosensors based on screen-printed electrodes were reported as encouraging analysis tools meeting the requirements of in situ screening devices, since all the equipment needed for the electrochemical analysis is portable. They have all the major performance characteristics of biosensors, such as good sensitivity, minimum sample preparation, simplicity of the apparatus and fast results readout. Moreover, they are cost effective, small and tend to be increasingly miniaturized thanks to new technologies [60].

The development of microfluidic lab-on-a-chip systems for analytical chemistry, biology, biomedical and clinical diagnostics applications has led to interesting analytical tools since these devices offer many potential advantages, including reduced reagent consumption, smaller analysis volumes, faster analysis times, and increased instrument portability [66].

## 4. Application of Electrochemical Biosensors Based on Enzyme Inhibition for Drug Screening

### 4.1. Acetylcholinesterase-Based Biosensors

Acetylcholinesterase (AchE) is considered as neurophysiological enzyme that controls the concentration of the neurotransmitter acetylcholine (Ach) in the synaptic cleft via the hydrolysis of acetylcholine into choline and acetate [67]. A decrease in concentration of acetylcholine in the brain has been associated with the memory loss observed in Alzheimer’s disease (AD). Thus, one approach in Alzheimer therapy is the use of acetylcholinesterase inhibitors that inhibit the hydrolysis of acetylcholine by acetylcholinesterase [68]. Reversible inhibitors of acetylcholinesterase such as tacrine, rivastigmine, neostigminen, pyridostigmine, donepezil, galantamine and eserine have all been used as therapeutic drugs for AD [69]. Therefore, the development of simplified devices with high sensitivity and fast response such as biosensors is needed in AD drug screening and in clinical diagnosis. Numerous cholinesterase inhibition-based biosensors have been developed for the detection of pharmaceutical compounds (Table 2).

Du et al. suggested in their work an electrochemical biosensor for the detection of galantamine and neostigmine. A homogenous film of silica sol-gel incorporating gold nanoparticles (AuNPs-Si-SG) was deposited electrochemically on a glassy carbon electrode. Then, acetylcholinesterase (AchE) was coated onto the modified glassy carbon electrode. The use of silica sol-gel with AuNPs not only prevents acetylcholinesterase from leaking out of the electrode surface but also creates a stable microenvironment for the enzyme activity [16].

Pohanka et al. proposed an electrochemical biosensor with acetylcholinesterase immobilized on a surface of the platinum working electrode. This biosensor was used to test two different drugs namely, tacrine and 7-MEOTA (the 7-methoxy derivative) used in treatment of cognitive manifestations of AD. The comparison of the inhibition effect of these two drugs revealed that 7-MEOTA is a 400 times stronger inhibitor than tacrine. This finding suggests the use of 7-MEOTA in treatment of AD cognitive manifestation at lower doses and with the same effect as tacrine, which make it a non-toxic alternative drug. This study showed that the biosensor technology could be used as a cheap and quick device for testing AchE inhibitors [18].

Parsajoo and Kauffmann developed a microfluidic device to monitor AchE activity and its inhibition. They successfully implemented a silver electrode for the amperometric detection of the artificial substrate thiocholine. They suggested that the developed system has a good sensitivity and selectivity towards compounds that react with silver chloride such as free thiol or sulfide. Furthermore, neostigmine inhibition effect was followed with the developed system in the range of 10^−8^–10^−5^ M with $I_{50}$ of 1.45 × 10^−7^ M [70].

Chemical mediators are widely used with screen-printed electrodes (SPE) to reduce the applied potentials, to increase the sensitivity of detection and to avoid interferences. Moreover, it is easy to incorporate the mediator in the ink for the preparation of screen-printed electrodes. Asturias-Arribas et al. developed AchE biosensor-based on screen-printed electrode (SPEs) using tetrathiafulvalene (TTF) as a mediator. A cross-linking method was used to immobilize AchE on TTF modified SPEs (TTF-SPEs). The suggested biosensor was optimized for chronoamperometric detection of codeine in urine samples and in pharmaceutical tablets. Codeine binds to AchE in a competitive way [71].

In another work, Zamfir et al. fabricated an AchE biosensor based on tetrathiafulvalene-tetracynoquinodimethane ionic liquid gel (TTF-TCNQ-IL). They characterized the biosensor using FT-IR spectroscopy and electrochemical impedance spectroscopy. This biosensor was used for the detection of carbamate drugs: neostigmine and eserine with limits of detection of 0.3 nM and 26 pM respectively. The proposed biosensor can be also applied in the screening of carbamates drug in wastewater [72].

Turan et al. reported a bi-enzymatic biosensor of acetylcholinesterase (AchE) and choline oxidase (ChO) based on graphite electrode coated with poly (BODT-co-FMOC) a conducting copolymer to monitor anti-dementia drugs (neostigmine and donepezil). They used the copolymer as an immobilization matrix for AchE and ChO. The authors used Scanning Electron Microscopy (SEM) and cyclic voltammetry as characterization techniques for the constructed biosensor. Neostigmine and donepezil inhibition effects on acetylcholinesterase were studied with the developed biosensor and the detection limits of 2.5 nM and 0.071 nM were reported respectively [73].

In their work, Vandeput et al. developed an amperometric biosensor based on immobilized acetylcholinesterase for the screening of several drugs known as AchE inhibitors. They used glutaraldehyde (GA) and cysteamine for a covalent immobilization of acetylcholinesterase on the surface of the gold microelectrode. They suggested that this type of immobilization allowed the retention of acetylcholinesterase activity, which facilitated the application of this system with reversible inhibitors of acetylcholinesterase [69].

For many years, magnetic particles (MPs) have been used as a good platform for the immobilization of enzymes through their attachment with amino, hydroxyl or carboxyl groups on the surface of MPs. Kostelink et al. suggested an acetylcholinesterase/MPs screen-printed electrode based biosensor for the detection of an anti-Alzheimer’s drug (galantamine). They immobilized acetylcholinesterase covalently using MPs and glutaraldehyde (GA). Under the optimized conditions, acetylcholinesterase inhibition by galantamine was studied in the range from 2.5 to 10 µM with a detection limit of 1.5 µM [74].

### 4.2. Monoamine Oxidase-Based Biosensors

Monoamine oxidase (MAO) catalyzes the production of hydrogen peroxide through the deamination of monoamine such as dopamine, adrenaline, serotonin, etc. This class of enzyme have two different isomers: monoamine oxidase A (MAO-A) and monoamine oxidase B (MAO-B) [75,76].

Monoamine oxidase inhibitors (IMAO) have shown potential uses in the treatment of several diseases such as depression and Parkinson’s disease [77]. The first monoamine oxidase inhibitors were clinically used as antidepressants. Since then, many derivatives of monoamine oxidase inhibitors were developed, with the ability to inhibit effectively, selectively and reversibly the enzymatic activity of MAO [78]. The most conventional technique used in drug control is chromatography [79]. However, this technique could be laborious and time consuming in contrary of biosensors that could be rapid, low cost and provide the required sensitivity needed in quantitative analysis of drugs.

In the last decade, the Medyantseva group have developed different amperometric biosensors based on immobilized MAO to monitor different drugs used as antidepressants [43,80,81,82,83,84,85,86] and nitrofuran drugs [87] in pharmaceutical tablets and urine samples (Table 3).

The first biosensor for analyzing pyrazidol, petylyl and fluoxetine (antidepressants) proposed by Medyantseva group was based on immobilized MAO with gelatin on platinum screen-printed electrode (SPE). These antidepressants act as inhibitors of MAO. The developed biosensor allowed the measurement of these inhibitors with low detection limits, which were 8 × 10^−7^ M, 8 × 10^−9^ M and 8 × 10^−10^ M for pyrazidol, petylyl and fluoxetine respectively [85,86].

Nitrofuran drugs are another class of drugs typically used as antibiotics and antimicrobials, which act as inhibitors of MAO. Volotskaya et al. suggested an amperometric biosensor based on immobilized MAO on screen-printed electrode for the assessment of nitrofuran drugs (furazolidone, furadonine and furagin) in pharmaceutical tablets and biological fluids such as urine. These drugs were assessed with the proposed biosensor with low detection limits of 8.5 × 10^−8^ M (furadonine), 8.3 × 10^−9^ M (furazolidone) and 9.4 × 10^−10^ M (furagin) [87].

In another work the Medyantseva group developed a MAO biosensor based on a screen-printed electrode (SPE) modified with multiwalled carbon nanotubes (MWCNTs) for determination of antidepressants. The monoamine oxidase (MAO) was immobilized on SPE by a cross-linking method using BSA and glutaraldehyde (GA). In this study, they used the developed biosensor to monitor the concentration of the antidepressants like imipramine, afobazole and phenazepam in drugs formulation and biological fluids (urine) [82,84].

On the other hand, the same group proposed a screen-printed electrode (SPE) modified with MWCNTs, silver nanoparticles (AgNPs) and immobilized MAO for determination of imipramine and amitryptline in urine. The inhibition effect of these antidepressants on MAO was studied in the concentration range of 10^−8^–10^−4^ M for both imipramine and amitryptline with detection limits of 7 × 10^−9^ M and 8 × 10^−9^ M respectively. The use of MWCNTs and AgNPs improved the sensitivity of the biosensor and extended the concentration range of the tested drugs [83].

In order to improve the analytical performance of MAO biosensor Brusnitsyn et al. suggested an amperometric MAO biosensor based on a screen-printed electrode (SPE) modified with nanomaterials such as carbon nanotubes (CNTs) and graphene oxide (GO) for monitoring antidepressants (moclobemide and amitriptyline) in pharmaceutical tablets [80]. The drugs used in this study were determined in the concentration range of 10^−8^–10^−4^ M.

Recently the Medyantseva group developed two different MAO biosensors modified with nanomaterials for monitoring moclobemide, tianeptine and amitriptyline antidepressants drugs in urine and in pharmaceutical tablets. In the first biosensor, they used screen-printed electrode modified with MWCNTs, gold and silver nanoparticles [43]. Using this biosensor, the antidepressants were determined in the concentration range of 5 × 10^−9^–10^−4^ M with a detection limit of 8 × 10^−10^ M. In the other biosensor, they modified screen-printed electrode with carbon nanotubes (CNTs) and AgNPs. These nanomaterials were stabilized by the use of functionalized hyperbranched polyesterpolyols [81]. The antidepressants drugs were tested with developed biosensor in a concentration range of 10^−8^–10^−4^ M and with a detection limit of 3 × 10^−9^ M.

Another group reported recently a MAO biosensor based on a SPE modified with manganese dioxide (MnO_2_) for measurement of selegiline hydrochloride, which is an irreversible inhibitor of MAO [1]. This inhibitor is used as drug in treatment of neurodegenerative diseases like Parkinson’s disease, Alzheimer’s disease and depression. Furthermore, the inhibitory effect of selegiline hydrochloride on immobilized MAO was followed in the concentration range of 2.2 × 10^−3^ M to 3.5 × 10^−4^ M with low detection limit of 0.67 × 10^−3^ M.

### 4.3. Xanthine Oxidase-Based Biosensors

Xanthine oxidase (XO) is an important enzyme that can catalyze both hypoxanthine and xanthine. It allows the oxidation of hypoxanthine (HYP) to xanthine (XAN), which in the next step is oxidized to uric acid (UA) as the final degradation product in the human purine catabolic pathway. The overproduction of these products lead to metabolic disorders such as xanthinuria, hyperuricemia, gout and renal failure [88,89]. The activity of this enzyme is strongly inhibited by allopurinol. Therefore, the use of allopurinol as an inhibitor of xanthine oxidase is one of the therapeutic strategies used to treat these metabolic disorders by blocking the production of uric acid. Allopurinol (1*H*-pyrazolo [3,4-d] pyrimidin-4-ol) is one of the xanthine oxidase inhibitors in clinical use to treat gout. Therefore, xanthine oxidase is the target enzyme in the implementation of biosensors to screen the drug allopurinol (Table 4).

A xanthine oxidase biosensing system was used for the first time for simultaneously monitoring allopurinol and oxypurinol at nanomolar concentrations by Hason and coworkers [90]. Allopurinol is oxidized by xanthine oxidase to oxypurinol, which remains tightly bound to the enzyme and ensures xanthine oxidase inhibition. The result obtained from the mentioned work confirmed that the inhibition of xanthine oxidase by allopurinol blocked the production of uric acid. Moreover, they applied the proposed biosensing electrochemical system to urine samples without any prior treatment for a simultaneous detection of metabolites involved in the xanthine oxidase pathway and excreted in urine [90].

A novel xanthine biosensor based on immobilization of xanthine oxidase by attractive materials layered double hydroxides was developed in 2009 [19]. Xanthine is a degradation product of purine derivatives, which at high concentrations in blood, plasma and urine may provide sensitive indicators of certain pathologic states, especially for xanthinuria. The developed biosensor was used for xanthine analysis by an amperometric detection at 0.55 V and the obtained linear response was ranging from 10^−6^ M to 2 × 10^−4^ M with a detection limit of 10^−7^ M. Using the same biosensor, xanthine oxidase inhibition by allopurinol was investigated and the obtained calibration curve was recorded in presence and in absence of the inhibitor. It was determined that allopurinol exerted a quasi-reversible competitive inhibition, which can compete with xanthine for the active site of xanthine oxidase to form the xanthine oxidase—inhibitor complex [19].

The side effects caused by synthetic drugs like allopurinol have prompted people to turn to traditional medicine using medicinal plants that are considered safer and more effective. Accordingly, many studies have been focused on the inhibitory effects of medicinal plants on xanthine oxidase [91,92,93,94,95]. As an example, a traditional medicinal plant named *Sida rhombifolia L.* has attracted increasing attention due to its anti-gout effects. The inhibition kinetics of *Sida rhombifolia L.* extracts toward xanthine oxidase were investigated using an electrochemical biosensing method [96]. Based on the obtained results, the inhibition type was determined to be competitive.

Recently, our group developed a simple and sensitive amperometric biosensor for the screening of medicinal plants for potential xanthine oxidase inhibitors [21]. In this work xanthine oxidase was immobilized for the first time on the surface of Prussian Blue-modified screen-printed electrodes using Nafion and glutaraldehyde. It was demonstrated that Prussian blue Deposited on the screen-printed electrodes has an excellent catalytic activity on the electroreduction of H_2_O_2_. The developed biosensor was tested first for allopurinol analysis. A linear range of allopurinol concentrations is obtained from 0.125 to 2.5 µM with an estimated 50% of inhibition $I_{50}=1.8\;µM$. On the other hand, the developed strategy was successfully applied in the screening of xanthine oxidase inhibitors from thirteen medicinal plants traditionally used by Moroccan people as infusions for the treatment of gout and its related symptoms. Moreover, the developed amperometric biosensor exhibited a good stability; it retained 90% of its initial activity for several weeks.

### 4.4. Cytochrome P450-Based Biosensors

Cytochrome P450s (CYPs) are a super-family of enzymes called hemoproteins due to the presence of a heme in their active site. They are involved in the metabolism of a variety of molecules such as drugs [97,98]. Thus, the study of metabolic enzyme inhibition is a crucial step in the investigation of drug toxicity and their development. In general, the assay of the catalytic activity of isolated cytochrome P450 family requires a redox couple (NADPH) [99]. The advantage of using electrochemical methods in the assay of cytochromes P450 is that their reduction occurs directly without need of electron donors. Therefore, the design of cytochrome P450 based biosensor may serve in the screening of a wide variety of drugs and the investigation of their inhibition or other effects. Table 5 summarized different isoforms of cytochrome P450 and the corresponding biosensors based inhibition.

Considerable efforts have been focused on the development of biosensors based on cytochrome P450 activity measurement. Many techniques have been used to improve the efficiency of these biosensors. To increase the electron transfer between the cytochrome P450 and the electrode, the use of different electrode type and the modification of surface transducers are of high relevance (Table 5).

Among different isomers of cytochrome P450, cytochrome P450-3A4 (CYP3A4) is the most used target enzyme in pharmaceutical fields as it metabolizes a majority of drugs [107,108]. Mie et al. investigated the inhibition of CYP3A4 by a drug called ketoconazole. CYP3A4 coupled with CYP reductase was immobilized on a naphthalenethiolate monolayer-modified gold electrode and effective direct electron transfer was observed. Electrochemical enzymatic reaction was carried out using testosterone as substrate. Upon the addition of ketoconazole, the cyclic voltammetry measurements showed a slight decrease in reduction current [100].

Carbon nanotubes (CNTs) and carbon nanofibers (CNFs) have attracted great interest recently as a new platform for biosensor assembly. The immobilization of a number of enzymes, including CYP enzymes, for the design of electrochemical biosensors using this new platform has been described [101,103].

Using a carbon nanofibers (CNFs)-based CYP3A4 biosensor the inhibition effect of ketoconazole was also reported [101]. The immobilization of CYP3A4 was achieved on a multilayer film to provide a suitable enzyme microenvironment and accelerate electron transfer. Carbon nanofibers (CNFs)-modified film electrodes were prepared on Si wafers fixed on plastic tape to construct disc electrodes. Excellent direct electron transfer was registered with the CYP3A4/CNFs-modified film electrode using both quinidine and testosterone as substrates. Using the developed biosensor, the inhibition effect of ketoconazole was assessed in the presence of testosterone as substrate and $I_{50}$ value of 58.5 mM was established. This result shows that ketoconazole is not a strong inhibitor of CYP3A4.

The use of nanomaterials such as multiwalled carbon nanotubes (MWCNT) in the design of cytochrome P450 biosensors improves the sensitivity, which allows one to reach low detection limits and consequently enhance drug screening and monitoring. Carrara group used multiwalled carbon nanotubes (MWCNT) for the design of cytochrome P450 (CYPs)-based biosensors to monitor multiple drugs [103]. For this purpose, various cytochrome P450 isoforms were used, including CYP 450 3A4, 2B4 and 2C9 for detecting different drugs: benzphetamine, cyclophosphamide (CP), dextromethorphan (DX), naproxen and flurbiprofen used as cough suppressant or in treatment of obesity, cancer and inflammation. The aim of this work was to emphasize the effect of drug mixtures on cytochrome P450 activity. Cyclic voltammograms obtained with CYP3A4 biosensor for the detection of cyclophosphamide in the presence of different concentrations of dextromethorphan up to 400 µM, showed that this drug inhibits the enzymatic catalysis of CYP3A4. They demonstrated in the aforementioned work that the use of MWCNT could improve the sensitivity of the method. Indeed, the limit of detection was enhanced from 59 to 12 µM in case of cyclophosphamide and from 187 to 82 µM in the case of naproxen. Consequently, the proposed biosensor should allow drug detection in human serum within the pharmacological range.

Later, MCNWTs were used for CYP3A4 sensor design. The enzymatic biosensor was employed for the study of abiraterone, a drug used in prostate cancer therapy [20]. The sensing system has been optimized and the investigations showed that the inhibition was irreversible when the concentration of abiraterone exceeded 1 µM. Recently, using the same biosensor, the same group managed the detection of abiraterone below micromolar range in human serum samples [104].

Baj-Rossi and coworkers modified a SPE surface with MWCNTs to immobilize microsomal CYP1A2 for naproxen detection. Naproxen (NAP) is non-steroidal anti-inflammatory drug widely used in osteo- and rheumatoid arthritis therapy. The proposed electrochemical biosensor was used for 16h NAP monitoring using continuous flow system and was calibrated to measure NAP in mouse serum [102]. The MWCNTs/CYP1A2-based biosensor can detect NAP in a linear range up to 200 µM with a detection limit of 16 µM. The interferences from real samples increase slightly the detection limit obtained with buffer.

The research team of Ajayi studied the competitive inhibition by paroxetine of CYP2D6 encapsulated in a nanostructured material [105]. Paroxetine, an antidepressant drug used in treatment of depression and panic disorders, showed an inhibition action on the fluvoxamine response on cytochrome P450-based biosensor. Poly (8-anilino-1-napthalene sulphonic acid) (PANSA) was deposited on a gold electrode by cyclic voltammetry and the enzyme was electrochemically encapsulated in the nanotubular structure of the Au-PANSA electrode. SEM microscopy showed that Au-PANSA nanotubes are organized in regular and uniform structures increasing the effective surface electrode to load large amounts of enzyme. Inhibition studies of paroxetine on CYP2D6-based biosensor was emphasized according to Dixon, Cornish Bowden and Lineweaver Burk Plots using paroxetine and fluvoxamine as inhibitor and substrate, respectively. The data obtained proved that paroxetine exhibited a competitive inhibition. The investigation on the CYP2D6-based Au-PANSA electrode showed a good analytical performance, excellent repeatability, short response times, long-term stability and low detection limit (0.002 μM paroxetine under mean plasma concentration). The biosensor recovered 40% of its activity by washing after a certain incubation time with paroxetine.

Another promising method to build an enzyme biosensor is based on the enzyme adsorption onto a dendrimer layer [106]. PANAM dendrimers offer better connectivity between gold electrodes and adsorbed enzymes. A quartz crystal microbalance was employed for adsorption of the PAMAM-Au nanoparticles and CYP3A4 ensuring the biosensor assembly. The inhibition test was monitored using caffeine as a cytochrome P450 substrate in the presence 10 mM erythromycin (as inhibitor). Cyclic voltammetry was conducted in the presence and absence of the inhibitor using the CYP3A4- based biosensor. Results showed a decrease in reduction current due to the introduction of erythromycin, which meant that the CYP3A4 activity was inhibited.

A new biosensor coupling DNA to enzymes was designed for the study of enzyme inhibition and consequently to prevent DNA damage due to CYP 450cam (CYP101) metabolism of styrene [17]. CYP/DNA film was assembled on a pyrolytic graphite (PG) electrode to construct the desired biosensor. The action of cytochrome P450 and imidazole inhibitors on the DNA damage were emphasized in this work. The apparent inhibition constant $K_{i}$ obtained from inhibition tests was of 268.2, 142.3 and 204.2 μM, imidazole, imidazole-4-acetic acid and sulconazole, respectively. Results showed a decrease in initial DNA damage rates with increasing inhibitor concentrations illustrating a successful application of CYP101/DNA biosensors.

### 4.5. Tyrosinase-Based Biosensors

Tyrosinase is an enzyme that holds two copper on its active site and catalyzes the production of *o*-quinones [109,110]. This enzyme has been used widely in the development of biosensors for the detection of phenolic compounds. Moreover, the inhibition of the tyrosinase activity was employed in the determination of toxic pollutants in environmental, biological samples [66,111,112] and drugs (Table 6).

Methimazole (MT) is one of the active drugs used in the therapy of hyperthyroidism. This drug inhibits the production of thyroid hormones due to its action that decreases the iodide incorporation into tyrosine. Therefore, recent electrochemical biosensors have been reported for the analysis of methimazole as an antithyroidal drug [23,24].

Martinez and coworkers presented and discussed an electrochemical biosensor based on tyrosinase inhibition for methimazole analysis. The resulting biosensor was constructed using a screen-printed electrode modified with carbon nanotubes and tyrosinase, providing a sensitive, fast and inexpensive analytical method. Using this method, methimazole determination was performed in the range of 0.074–63.5 µM with a detection limit of 0.056 µM [24]. The addition of methimazole caused a decrease in the reduction current of *o*-benzoquinone due to the formation of the corresponding thioquinone derivatives. Furthermore, the application of this biosensor to different pharmaceutical samples containing methimazole supports the utility of this method.

Another biosensor was developed by Kurbanoglu and coworkers, for the investigation of methimazole, using a nanoparticles based lab-on-a-chip system [23]. In this work, an amperometric biosensor was designed using magnetic nanoparticles decorated with iridium oxide nanoparticles and tyrosinase immobilized onto a screen-printed electrode. The developed biosensor is based on tyrosinase inhibition: as tyrosinase loses copper, the conformation of the enzyme changes and irreversible inhibition occurs. A methimazole evaluation has been done in this work using both batch and lab-on-a-chip microsystems with integrated screen-printed electrodes. The obtained chronoamperometric results confirmed that both systems are very sensitive. The limit of detection of methimazole was 0.006 μM and 0.004 μM for batch and flow modes, respectively. On the other hand, the flow mode has many good features such as its reusability, automation, fast response (20 s) and low sample volume (6 μL). The performed methods have been applied in methimazole analysis in spiked human serum and pharmaceutical dosage forms.

Pipemidic acid (PA) is a synthetic quinolone drug used as an antibacterial agent. Bertolino and coworkers developed a microfluidic-enzymatic sensor combined with electrochemical detection designed for the quantification of pipemidic acid [66]. The detection was performed using tyrosinase biosensor-based inhibition. In this work, the enzyme was immobilized on 3-aminopropyl-modified-controlled-pore glass packets in a central channel of a microfluidic-enzymatic device. After the enzymatic oxidation of catechol to *o*-benzoquinone, the electrochemical reduction of *o*-benzoquinone was detected at a gold electrode surface at 0 V vs. Ag/AgCl before and after enzyme inhibition by pipemidic acid. Upon the addition of pipemidic acid, the reduction current decreases due to the formation of amino-quinone derivatives. The usefulness of this microfluidic-enzymatic sensor coupled with electrochemical method toward the detection of pipemidic acid at very low concentrations in pharmaceutical samples has been confirmed in this work. The recovery of pipemidic acid from four pharmaceutical samples ranged from 97.50% to 102.50%. The proposed method could be used to determine the pipemidic acid concentration in the range of 0.02–70 µM with a limit of detection of 18 nM.

A novel biosensor based on the inhibition of tyrosinase activity by captopril, which is generally used to treat congestive heart failure, has been developed by Kurbanoglu and coworkers [113]. The designed biosensor is based on a screen-printed electrode modified with electrochemically reduced graphene oxide and iridium oxide nanoparticles for the detection of the angiotensin-converting enzyme inhibitor drug. In this work tyrosinase was immobilized onto the modified screen-printed electrode using 1-ethyl-3-(3-dimethylaminopropyl)-carbodiimide and N-hydroxysuccinimide coupling reagents. They demonstrated that captopril detection using two different inhibition pathways are very sensitive. They achieved low detection limits equal to 0.019 μM and 0.008 μM for the inhibition of the chelation of copper at the active site of tyrosinase and thioquinone formation in presence of captopril, respectively.

Many researchers have linked the presence of protease to diseases (e.g., AIDS, neurodegenerative diseases, cancer) [114,115,116]. Recently, our group proposed a new and a sensitive approach to assay protease activity and inhibition using a tyrosinase biosensor [22]. The biosensor was elaborated using carbon nanopowder electrode modified with tyrosinase. The proposed approach was based on the electrochemical detection of tyrosine liberated from the hydrolysis of a protein (casein) by a protease. The use of tyrosinase biosensor enhanced the sensitivity and allowed the assay of protease activity at very low level. Leupeptin is a natural inhibitor of protease and reported as a potential drug for cancer [117,118]. This inhibitor was assessed in the linear range of 0.25 to 5 µM with a detection limit of 0.25 µM (Table 6). Furthermore, the results showed an uncompetitive and reversible binding of leupeptin with protease.

### 4.6. Other Enzyme Inhibition-Based Biosensors

Among several drugs, nonsteroidal anti-inflammatory drugs (NSAID) are widely used against several diseases, especially arthrosis since they compete with arachidonic acid that binds to cyclooxygenase isoforms (COx1 and COx2). COx1 exists all over the human biological system, while COx2 is rarely present in the stomach. The cyclooxygenase-based biosensor have been described for the detection of NSAIDs drugs including naproxen, diclofenac, ibuprofen and tolmetin [119]. The enzymatic activity yielded by the biosensor was followed by the measurement of oxygen concentration using arachidonic acid as a substrate. The COx1 and COx2 was immobilized in a gel- like membrane fixed at the PTFE cap of a Clark electrode to elaborate two different biosensors. The results showed that both devices have similar performance properties, except for the longer lifetime characteristic observed for the COx1-based electrode. The reported limits of detection of the tested drugs were 5.0 × 10^–8^ M for naproxen and diclofenac and 0.5 × 10^–8^ M for ibuprofen and tolmetin (Table 7).

DNA methylation phenomena are correlated with various diseases, especially cancer. Aberrant methyltransferase enzyme (MTase) is associated to the pathogenesis of leukemia. Therefore, the study of the MTase activity and its inhibitors is important in the cancer curing process. Yin and coworkers [120] developed a novel screening strategy for drugs as enzyme inhibitors using DNA methyltransferase-based biosensors. Successive hybridization steps of DNA strands conjugated to Au-NPs were realized for the design of this biosensing system. The electrochemical signal measured is due to the response of ferrocenecarboxylic acid-functionalized DNA strands attached to Au-NPs. The inhibition of DNA methyltransferase by fisetin was carried out after digestion by HpaII endonucleases. Differential pulse voltammetry was applied to screen the inhibition effect of different concentration of fisetin in the micromolar range.

Aberrant O-linked N-acetylglucosamine transferase OGT activity has been found to be involved in many diseases such as diabetes [121], Alzheimer’s disease [122] and cancer [123,124,125]. A label-free electrochemical biosensing device was elaborated using protease to monitor peptide O-GlcNAcylation [126]. Synthetized peptide containing free tyrosine residues was immobilized on the surface of ITO electrode (on the N terminal) and later glycosylated by O-linked N-acetylglucosamine (O-GlcNAc) transferase (OGT). Using the proposed biosensor, the introduction of protease activity could distinguish between glycosylated and non-glycosylated peptides. When the O-GlcNAcylation is successfully inhibited using a small molecule, the unglycosylated peptides can be cleaved easily which led to low current signals. The oxidation current of tyrosine decreases with increasing inhibitor concentrations. BZX (phenyl 5-chloro-2-oxo-3-hydrobenzoxazole-3-carboxylate) and alloxan, known potent OGT inhibitors, were used for simple inhibition assays. Further work was proposed by the authors to develop a microarray OGT inhibition-based biosensor format and its application to real samples under different conditions.

Diabetes is one the most common diseases in the world. In traditional medicine, the use of natural plants with anti-diabetic activities is widely used in the treatment of type 2 diabetes mellitus, called also non-insulin-dependent diabetes (NIDDM). The biological activity of these natural compounds was verified by inhibition of α-glycosidase (AG). In 2007, an electrochemical biosensor was used for the detection of two different drugs (amaryl and acarbose) that act as α-glycosidase inhibitors [127]. The biosensor consists on an α-glycosidase and gelatin membrane immobilized via cross-linking by glutaraldehyde on a bi-film modified glassy carbon electrode (Table 7). The enzyme activity was followed using *p*-nitrophenyl α-D-glucopyranoside (PNPG) which is converted to 4-nitrophenol and measured by chronoamperometry. The results showed that the inhibition of α-glycosidase (AG) with amaryl and acarbose was reversible. Therefore, it will be convenient to use the developed biosensor for the screening of medicinal plants having the ability to inhibit α-glycosidase.

Furthermore, α-glycosidase enzyme was immobilized onto an amine-functionalized multiwalled carbon nanotubes (MWCNTs-NH_2_)-based electrode [128]. The biosensor was applied in the screening of potential inhibitors from medicinal plant extracts that have an anti-diabetic action. The novel approach in this work was based on the entrapment of the enzyme in a polymer (polyvinyl alcohol) together with the *p*-nitrophenyl α-D-glucopyranoside on a screen-printed carbon electrode at low pH to prevent the premature reaction between substrate and enzyme. In the presence of *Tebengau* plant extracts, the α-glycosidase enzymatic activity was inhibited, suggesting the application of the developed biosensor in the rapid screening of inhibitors from medicinal plants, which will prevent the enzymatic production of glucose.

Sulfonamides (SA’s) are a superfamily of drugs used in human and veterinary medicine. In the body, they inhibit carbonic anhydrase enzyme. The inhibition reaction can be used as tool for the detection of SA’s pharmaceutical residues in biological and environmental samples. Our research group developed an electrochemical carbonic anhydrase (CA)-based biosensor for the detection of sulfanilamide (SAD) [129]. The enzyme was incorporated into a carbon paste electrode prepared with carbon black nanoparticles. The SAD measurement was realized by following CA inhibition using linear sweep voltammetry. The enzymatic activity was studied by monitoring the conversion of 4-nitrophenylacetate (NPA) into 4-nitrophenol in the absence and presence of SAD. The estimated detection limit achieved by the CA-based biosensor was equal to 0.4 µM (Table 7). The sulfanilamide exhibits a noncompetitive inhibition on CA enzyme according to Lineweaver-Burk representation.

## 5. Conclusions and Perspectives

This review compiles research activities realized during the last decade using electrochemical biosensors for drug detection based on enzymatic inhibition. The usefulness and the importance of these biosensors in analytical biochemistry for the screening of drugs and their implication in the therapy of various diseases were reported. Moreover, since the phenomena of enzyme inhibition is unavoidably associated with drug therapy, the inhibition mechanisms were also discussed.

Some of the most important steps in drug-metabolizing phenomena are based on enzymatic reactions. The enzymes most involved in this biotransformation include CYP enzymes. Other enzymes, including acetylcholinesterase, xanthine oxidase, monoamine oxidase, etc., are considered as drug targets. In general, the consequences of prescribing drugs may result on enzymatic inhibition.

In this paper, we focus our review on monitoring enzyme inhibition for pharmaceutical and clinical applications using electrochemical biosensors. Aberrant activity of some enzymes is associated with various diseases. Direct measurement of enzyme activity in biological samples before and after administration of drug is an obvious method for assessing inhibition effects, among others, before proceeding to in vivo novel drug administration studies. Numerous drugs are analyzed in this way, such as antitumoral, anti-epilepsy, anti-infectious agents, etc. Therefore, it is important to develop a simple and sensitive method for enzymatic activity assays. More importantly, for curing diseases caused by aberrant enzymes, the screening of drugs as enzyme inhibitors is also necessary and interesting.

The electrochemical biosensors based on enzyme inhibition are more and more used in drug screening as alternative techniques to conventional methods. They are simple, miniaturized, fast and sensitive. For all these reasons and others, the present review reports the latest electrochemical biosensors designed in the clinical and pharmaceutical field. The enzymes employed, inhibition type induced by various drugs, the transducers used and their applications were discussed. The recent works reported in this review indicate the potential applications of biosensors based on enzyme inhibition for drug screening and monitoring. Hence, more attention should be focus on the application of biosensors on real samples and clinical cases. Accordingly, biosensor development should be more focused on miniaturized, sensitive and portable devices for analyzing real samples.

In addition, this review showed that in the inhibition studies, the kinetics of enzymatic reaction in absence and presence of different inhibitors are poorly reported. The novel graphical approach proposed a few years ago by Amine et al. [48] can be used as an effective tool for detailed inhibition investigations. The developed graphical method helps distinguish between different types of reversible inhibition by measuring $I_{50}$ using just two different substrate concentrations. The $I_{50}$ versus substrate concentration changes from competitive to uncompetitive inhibition. In non-competitive inhibition, substrate concentration does not affect the $I_{50}$. Moreover, the graphical method helps also to distinguish between reversible and irreversible inhibitors as pointed out in this review. Indeed, in irreversible inhibition, the $I_{50}$ is affected by the incubation time and enzyme concentration at the opposite of reversible inhibition where does not change.

The personalized screening methods such as biosensors based on enzyme inhibition are a promising technology in the pharmaceutical and clinical fields, especially to improve the quality of life of patients with several diseases and subjected to numerous drug therapies.

## Figures and Tables

**Table 1:** Summary of some drug-target enzyme and related diseases.

Target Enzyme	Inhibitor/Drug	Inhibition Type	Clinical Use	Reference
Xanthine oxidase	Allopurinol	Quasi-reversible	Gout	[27]
Carbonic anhydrase	Acetazolamide	Reversible	Glaucoma	[28]
Monoamine oxidase A	Moclobemide	Reversible	Depression	[29]
Monoamine oxidase A	Transylcypromine	Irreversible	Depression	[29]
Monoamine oxidase B	Selegiline	Irreversible	Parkinson’s disease	[29]
Acetylcholinesterase	Donepezil	Reversible	Alzheimer’s disease	[30]
			Autism	
Acetylcholinesterase	Galantamine	Reversible	Alzheimer’s disease	[30]
	Tacrine			
	7-methoxytacrine			
Acetylcholinesterase	Rivastigmine	Reversible	Alzheimer’s disease	[30]
			Parkinson’s disease	
GABA Transaminase	Vigabatrin	Irreversible	Epilepsy	[31]
Thrombin	Argatroban	Reversible	Cardiovascular disease	[32]
Cyclooxygenase	Aspirin	Irreversible	Inflammation, Pain, Fever	[33]
β-lactamase	Clavulanate	Irreversible	Bacterial resistance	[34]
Viral DNA polymerase	Acyclovir	Reversible	Herpes	[35]
Viral neuraminidase	Relenza	Reversible	Influenza	[36]
HIV-1 Protease	Ritonavir	Reversible	AIDS	[37]
HIV-1 Protease	Zidovudine	Irreversible	AIDS	[38]
Tyrosine-Kinase	Gefitinib	Reversible	Cancer	[39]
Angiotensin converting enzyme	Captopril	Reversible	Hypertension	[40]
	Enalapril			
DNa gyrase	Norfloxacin	Reversible	Urinary tract infection	[41]

**Table 2:** Summary of acetylcholinesterase inhibition based biosensors.

Transducer	Inhibitor (Drug)	Detection Method	Detection Limit	Sample	References
GCE/AuNPs-Si-SG/AchE	Galantamine	Cyclic voltammetry and amperometry	-	-	[16]
	Neostigmine				
Pt/AchE	Tacrine	Chronoamperometry	-	-	[18]
	7-Methoxy-Tacrine (7-MEOTA)				
Flow-through sensor/AchE	Neostigmine	Amperometric flow injection	-	-	[70]
SPE/TTF/AchE	Codeine	Chronoamperometry	-	Urine and pharmaceutical tablets	[71]
TTF-TCNQ-IL/AchE	Neostigmine	Chronoamperometry	0.3 nM (Neostigmine)	Tap water	[72]
	Eserine		26 pM (Eserine)		
GE/BODT-co-FMOC/Cho/AchE	Neostigmine	Amperometry	2.5 nM (Neostigmine)	Tap water	[73]
	Donepezil		0.071 nM (Donepezil)		
Flow-through sensor/AchE	Neostigmine	Amperometric flow injection	-	-	[69]
	Eserine				
	Tacrine				
	Donepezil				
	Rivastigmine				
	Pyridostigmine				
	Galantamine				
SPE/MPs/AchE	Galantamine	Square wave voltammetry	1.5 µM	-	[74]

AuNPs: Gold nanoparticles; Si-SG: Silica sol-gel; TTF: Tetrathiafulvalene; TCNQ-IL: Tetracyanoquinodimethane ionic liquid gel; BODT-co-FMOC: 5,6-bis(octyloxy)-4,7-di(thiophen-2-yl)benzo[c][1,2,5]oxadiazole(BODT) with (2-(((9H-fluoren-9-yl)methoxy)carbonylamino) acetic acid (FMOC); SPE: Screen-printed electrode; MPs: Magnetic particles; AchE: Acetylcholinesterase.

**Table 3:** Summary of monoamine oxidase inhibition based biosensors.

Biosensor	Inhibitor (Drug)	Concentration Range	Limit of Detection	Sample	References
Pt-SPE/MAO	Petylyl	10^−8^ M–10^−4^ M	8 × 10^−9^ M		[85]
	Pyrazidol	10^−7^ M–10^−4^ M	8 × 10^−7^ M		
	Fluoxetine	10^−9^ M–10^−4^ M	8 × 10^−10^ M		
Pt-SPE/MAO	Furazolidone	10^−8^ M–10^−4^ M	8.3 × 10^−9^ M	Urine and pharmaceutical tablets	[87]
	Furadonine	10^−7^ M–10^−5^ M	8.5 × 10^−8^ M		
	Furagin	10^−9^ M–10^−5^ M	9.4 × 10^−10^ M		
SPE-MWCNTs/MAO	Phenazepam	10^−9^ M–10^−4^ M	6 × 10^−10^ M	Urine and pharmaceutical tablets	[82]
	Imipramine		8 × 10^−10^ M		
	Afobazole		9 × 10^−10^ M		
SPE-CNTs-GO/MAO	Moclobemide	10^−8^ M–10^−4^ M	5 × 10^−9^ M	Pharmaceutical tablets	[80]
	Amitriptyline		8 × 10^−9^ M		
SPE-MWCNTs-AuNPs-AgNPs/MAO	Moclobemide	5 × 10^−9^ M–10^−4^ M	8 × 10^−10^ M	Urine and pharmaceutical tablets	[43]
	Tianeptin	10^−8^ M–10^−4^ M	7 × 10^−9^ M		
SPE-MnO_2_/MAO	Selegiline	2.2 × 10^−3^ M–3.5 × 10^−4^ M	0.67 × 10^−3^ M	Pharmaceutical tablets	[1]
	Hydrochloride				

MWCNTs: Multi-walled carbon nanotubes; AgNPs: silver nanoparticles; CNTs: Carbon nanotubes; GO: Graphene oxide; AuNPs: Gold nanoparticles; MnO_2_: Manganese dioxide.

**Table 4:** Summary of different amperometric xanthine oxidase based biosensors used for inhibition studies.

Transducer	Detection Method	Inhibitor (Drug)	Linear Range/$I_{50}$	Sample	Reference
Pt-LDHs/XO	Amperometry	Allopurinol	10 to 800 µM	-	[19]
			$I_{50}=310\;µM$		
SPE-PB/Nafion/XO	Amperometry	Allopurinol	0.125–2.5 µM	medicinal plants	[21]
			$I_{50}=1.8\;µM$		

Pt: Platinum electrode; LDHs: materials layered double hydroxides; SPE: screen-printed electrode; PB: Prussian blue; XO: xanthine oxidase.

**Table 5:** Summary of different CYP isoforms and corresponding biosensors used for inhibition studies.

Transducer	Detection Method	Inhibitor (Drug)	$I_{50}$/$K_{i}$	Reference
Au-NT/CPR/CYP3A4	CV	Ketoconazole	-	[100]
CNF/CYP3A4	CV	Ketoconazole	$I_{50}$ = 58.5 mM	[101]
MWCNT/CYP1A2	CV	Naproxen	-	[102]
		Linear range: 50–300 µM		
		(LOD = 16 µM)		
MWCNT/CYP3A4	CV	Dextromethorphan	-	[103]
MWCNT/CYP3A4	CV	Abiraterone (irreversible)	-	[20]
		linear range:1–3 µM		
MWCNT/CYP3A4	CV	Abiraterone	-	[104]
		(LOD = 230 nM)		
		Dynamic range up to 1 µM		
Au-PANSA/CYP2D6	DPV	Paroxetine (reversible)	$K_{i}=0.02$ µM	[105]
	CA	linear range: 0.005–0.05 µM	$I_{50}$ = 0.03 µM	
	SWV	(LOD = 2 nM)		
Au-NPs-PANAM dendrimers/CYP3A4	CV	Erythromycin	-	[106]
PG/DNA/CYP101	SWV	Sulconazole	$K_{i}=204.2$ μM	[17]

NT: naphtalenethiolates; Au: gold electrode; CPR: Cytochrome reductase; CNF: Carbon nanofibers; MWCNTs: multiwalled carbon nanotubes; PANSA: Poly(8-anilino-1-napthalene sulphonic acid); PAMAM: Polyamido-amine; PG: Pyrolitic graphite; CV: Cyclic voltammetry; SWV: Square Wave Voltammetry; DPV: differential pulse voltammetry; CA: chronoamperometry.

**Table 6:** Summary of amperometric tyrosinase based biosensors used for inhibition studies.

Transducer	Inhibitor (Drug)	Detection Method	Linear Range/LOD	Sample	References
SPE-CNTs/Tyr	Methimazole	Amperometry	0.074–63.5 µM	Commercial preparations	[24]
			LOD = 0.056 µM		
SPE-Irox NPs/Tyr/MNPs	Methimazole	Amperometry	0.1–10 µM	Spiked human serum and pharmaceutical dosage forms	[23]
	(Irreversible)		LOD = 0.006 μM		
APCPG/Tyr	Pipemidic acid	Cyclic voltammetry	0.02–70 µM	Pharmaceutical samples	[66]
			LOD = 18 nM		
ERGO/IrOxNPs/Tyr	Captopril	Amperometry	0.1–15 µM	Spiked human serum and pharmaceutical dosage forms	[113]
			LOD = 0.019 μM		
			0.05–14 µM		
			LOD = 0.008 μM		
CNPE/Tyr	Leupeptin	Chronoamperometry	0.25–5 µM	-	[22]
	(Reversible)		LOD = 0.25 µM		

CNTs: Carbon nanotubes; APCPG: 3-aminopropyl-modified controlled-pore glass; Tyr: Tyrosinase; SPE: screen-printed electrode; Irox NPs: iridium oxide nanoparticles; MNPs: magnetic nanoparticles; ERGO: Electrochemically reduced graphene oxide; IrOxNPs: iridium oxide nanoparticles; CNPE: carbon nanopowder paste electrode.

**Table 7:** Summary of other enzymes and corresponding biosensors used for inhibition studies.

Biosensor	Inhibitor/Drug	Detection Method	Linear Range/LOD	Sample	Reference
Clark electrode/COx	Naproxen	Amperometry	Naproxen and Diclofenac LOD (5 × 10^−8^ µM)	Pharmaceutical Drug	[119]
	Diclofenac				
	Ibuprofen		Ibuprofen and Tolmetin LOD (0.5 × 10^−8^ µM)		
	Tolmetin				
SPE-MWCNT-NH2/AG-PNPG	Tebangu plant extracts	Amperometry	0.423–8.29 µM	-	[128]
		Cyclic Voltammetry	LOD (0.253 µM)		
GCE/GE/AG	Amaryl	Chronoamperometry	-	-	[127]
	Acorbose (Reversible)	Cyclic Voltammetry			
CPE/CA	Sulfanilamide (Reversible)	Linear Sweep Voltammetry	0.5–5 µM	-	[129]
			LOD (0.4 µM)		

CPE: carbon paste electrode; Cox: cyclooxygenase; AG: α-glucosidase; PNPG: *p*-nitrophenyl-α-D-glucopyranoside; MWCNTs-NH_2_: amine-functionalized multi-walled carbon nanotubes; SPE: screen-printed electrode; GE: Gelatine; GCE: glassy carbon electrode; CA: Carbonic anhydrase.
